# CRISPR/Cas9-mediated gene mutation of *EcIAG* leads to sex reversal in the male ridgetail white prawn *Exopalaemon carinicauda*

**DOI:** 10.3389/fendo.2023.1266641

**Published:** 2023-11-21

**Authors:** Miao Miao, Shihao Li, Jianbo Yuan, Peipei Liu, Xiaochen Fang, Chengsong Zhang, Xiaojun Zhang, Fuhua Li

**Affiliations:** ^1^CAS and Shandong Province Key Laboratory of Experimental Marine Biology, Institute of Oceanology, Chinese Academy of Sciences, Qingdao, China; ^2^University of Chinese Academy of Sciences, Beijing, China; ^3^Center for Ocean Mega-Science, Chinese Academy of Sciences, Qingdao, China; ^4^The Innovation of Seed Design, Chinese Academy of Sciences, Wuhan, China

**Keywords:** insulin-like androgenic gland hormone, *Exopalaemon carinicauda*, CRISPR/Cas9, sex reversal, gene editing

## Abstract

In the culture of crustaceans, most species show sexual dimorphism. Monosex culture is an effective approach to achieve high yield and economic value, especially for decapods of high value. Previous studies have developed some sex control strategies such as manual segregation, manipulation of male androgenic gland and knockdown of the male sexual differentiation switch gene encoding insulin-like androgenic gland hormone (IAG) in decapods. However, these methods could not generate hereditable changes. Genetic manipulation to achieve sex reversal individuals is absent up to now. In the present study, the gene encoding IAG (*EcIAG*) was identified in the ridgetail white prawn *Exopalaemon carinicauda*. Sequence analysis showed that *EcIAG* encoded conserved amino acid structure like IAGs in other decapod species. CRISPR/Cas9-mediated genome editing technology was used to knock out *EcIAG*. Two sgRNAs targeting the second exon of *EcIAG* were designed and microinjected into the prawn zygotes or the embryos at the first cleavage with commercial Cas9 protein. *EcIAG* in three genetic males was knocked out in both chromosome sets, which successfully generated sex reversal and phenotypic female characters. The results suggest that CRISPR/Cas9-mediated genome editing technology is an effective way to develop sex manipulation technology and contribute to monosex aquaculture in crustaceans.

## 1 Introduction

Crustaceans are important economic species in aquaculture, which usually exhibit sexual dimorphism (1, 2). Sexual dimorphism leads to different morphological, physiological and behavioral characteristics between males and females (3, 4), which exhibit sex differences in their biology and economic value (5, 6). Some species showed that males have larger body length and body weight than females (7), while in some other species, females shows faster growth rates and larger body size compared with males (8). Monosex culture is not only beneficial to increase the yield, but also has ecological significance to avoid the invasive risk of cultured species (1, 9). All-male cultures of *Macrobrachium rosenbergii* is much more profitable than mixed and the all-female cultures (10). The risk of leakage into natural waters is reduced when exotic species of only one sex are introduced because they cannot reproduce (11).

The methods of obtaining monosex populations are usually manual segregation and sex manipulation (11). However, manual segregation is a labor-intensive and cumbersome method (10). Sex manipulation is an effective and beneficial technique in obtaining monosex populations (11). Understanding the mechanism of sex determination and differentiation is the key to sex manipulation.

The sex determination and sexual differentiation in crustaceans were regulated by multiple genes (12). Numerous sex-related genes have been identified in crustaceans, such as insulin-like androgenic gland hormone (IAG), crustacean female sex hormone (CFSH), Wnt4, Dmrt gene family, Sox gene family, Fem-1, etc (13). IAG and CFSH are two important hormones involved in sexual differentiation. CFSH plays an essential role in controlling the female differentiation, while the sexual differentiation of males is regulated by IAG secreted by the androgenic gland (AG) (14). AG is a unique organ involved in the differentiation of the male gonad and the maintenance of secondary sex characteristics (15–17). IAG is regarded as an “IAG-switch” of the X-organ-sinus-gland (XO-SG)-androgenic gland (AG)-testis regulatory axis in male crustacean (18). IAG expression could induce masculinization, while AG ablation or IAG suppression leads to feminization. IAG belongs to the insulin-like peptide superfamily, whose structure is highly conserved in crustaceans (19). So far, the IAG gene has been identified in dozens of decapod crustaceans, including prawns, shrimp, crayfish, lobsters, and crabs (20).

Extensive research on sex determination and differentiation has provided theoretical support and targets for sex manipulation. At present, sex manipulation in decapod crustaceans is performed mainly through AG manipulation and the suppression of sex-related genes by RNAi. The neo-females of *Macrobrachium rosenbergii* were acquired by AG ablation, the neo-males were acquired by AG implantation (7, 21). Silencing of *MrIAG* and *MroDmrt11E* also induced complete and functional sex reversal of *M. rosenbergii* (22, 23). In the Australian red-claw crayfish, implantation of AG led to the appearance of masculine behavior (24). However, no inheritable germplasm materials were obtained for sex control in crustaceans till present.

CRISPR/Cas9-mediated genome editing technology enables direct modification of genes at the genome level (25). It has been applied to gene function studies in many crustaceans. In *Daphnia magna*, the CRISPR/Cas9 system induced heritable mutations into the *eyeless* gene (26). In *Exopalaemon carinicauda*, the functions of chitinase 4 (*EcChi4*), two homologs of Paired box protein 6 (Pax6) and a DILP7/relaxin-type insulin-like peptide (*EcILP*) were verified by the CRISPR/Cas9-mediated genome editing technology (27–29). In addition, the CRISPR/Cas9-mediated genome editing technology have also been successfully used in *Neocaridina heteropoda*, *Eriocheir sinensis*, and *Macrobrachium rosenbergii* (30, 31). Therefore, sex manipulation by editing the IAG gene directly on the genome could be an efficient way to generate inheritable monosex populations. However, there are still some defects in the application of CRISPR/Cas9-mediated genome editing technology in crustaceans, such as low editing efficiency, lack of biallelic mutants and adult mutants (32, 33).

The ridgetail white shrimp, *E. carinicauda*, is a kind of crustacean widely distributed in the coasts of eastern China and western Korea (34). As an economic prawn species, it has many advantages, such as rapid growth, good reproduction capability and strong ability to adapt to environment (35). This species shows apparent sexual dimorphism (36). Its breeding cycle is about 60 days. Although the number of eggs is relatively small, the size of the egg is moderate, which is easy to operate by gene editing microscopy (37, 38). In this study, the *EcIAG* was identified from *E. carinicauda*. *EcIAG* was knocked out by CRISPR/Cas9 genome editing technology and the sex reversal neo-female individuals with phenotypic female characters were obtained. This data provided a possibility for suppressing male sexual differentiation at the genome level and applying gene editing technology for monosex breeding in crustaceans.

## 2 Materials and methods

### 2.1 Breeding and hatching of *E. carinicauda*

The ridgetail white prawn used in genome editing were raised on a farm in Huanghua City, Hebei Province. The sexually mature female and male prawns were selected artificially and cultivated in a tank containing 100 liters of aerated sea water at 26 ± 1 °C. After spawning, the embryos of *E. carinicauda* were collected from the abdomen of gravid female prawns into petri dishes containing filtering seawater. The number of nuclei was observed under a stereomicroscope, and the zygotes or the embryos at the first cleavage were selected for microinjection. Then, the selected embryos were stored at 12 °C before microinjection to retard the speed of development.

The ridgetail white prawns used for cloning the full-length cDNA of *EcIAG* were cultivated in our laboratory, which were from a full-sib family with the ridgetail white prawns on the farm.

### 2.2 Screening of *EcIAG* in *E. carinicauda*

Based on the genome and transcriptome data of *E. carinicauda* in our laboratory (37), the *EcIAG* was searched by gene functional annotation and description. Furthermore, the nucleotide sequences of IAG and its deduced amino acid sequences were compared to the sequences of known IAGs from other different crustaceans using the BLAST algorithm (https://blast.ncbi.nlm.nih.gov/Blast.cgi) to identify potential *EcIAG* sequence with an E-value cutoff of 1e^-5^. The open reading frame (ORF) of candidate *EcIAG* sequence was predicted using ORF Finder (https://www.ncbi.nlm.nih.gov/orffinder/).

### 2.3 Cloning of the full-length cDNA of *EcIAG*

Total RNA was extracted from the AG of *E. carinicauda* using RNAiso plus reagent (Takara, Kyoto, Japan) according to the manufacturer’s protocol. The purity and concentration of RNA were qualified by 1% agarose gel electrophoresis and Nanodrop 2000 spectrophotometer (Thermo Fisher Scientific, Waltham, MA, USA). Then, total RNA (1 μg) was reverse-transcribed into cDNA using the PrimeScript™ RT Reagent Kit with gDNA Eraser (TaKaRa, Kyoto, Japan) to remove the genomic DNA. Specific primers used to amplify the *EcIAG* cDNA were designed by Primer-BLAST (https://www.ncbi.nlm.nih.gov/tools/primer-blast/) (**Table 1**). The full-length cDNA of *EcIAG* was amplified by polymerase chain reaction (PCR) using PrimeSTAR^®^ GXL DNA Polymerase (TaKaRa, Kyoto, Japan). Then, the product of PCR was ligated into the pMD19-T vector (TaKaRa, Kyoto, Japan) and transformed into trans5α competent cells (TransGen Biotec, Beijing, China). The monoclones were selected and sequenced at Sangon Biotech Co., Ltd. (Shanghai, China). The sequenced ORF was mapped the genomic data to identify the exons and introns using the NCBI BLAST.

**Table 1:** Primers used in this paper.

Primers	Sequences (5’-3’)	Annealing temperature (°C)
Full-length primer		
EcIAG-cF	TCTCTGTCTCTCTCAGCTTG	58
EcIAG-cR	CTAGCTCTCCGGAACGGCAGG	
Evaluation primer		
EcIAG-eF	TAGCTGTAAGTGCAGGTAGAA	57
EcIAG-eR	CTGCATCACTAACTTTTGGTAAT	
Sex identification primer		
EcSDF	TCACACAGAATGGATGCAACT	60
EcSDR	TCCATGGGTTTGATCAATCCCT	

### 2.4 Sequence characterization of *EcIAG*

To know the evolution of *EcIAG*, multiple sequences alignment and neighbor-joining (NJ) phylogenetic analysis with 1000 bootstrap replicates were conducted by MEGA version 7.0 (39).The amino acid sequences of IAG homologs from other crustaceans were downloaded from the NCBI database. The similarity of *EcIAG* from different species was acquired from NCBI BLASTP alignments. And the ExPASy (Expert Protein Analysis System, https://www.expasy.org) was used to analyze the deduced amino acid sequence.

### 2.5 sgRNA design and synthesis

The single-stranded guide RNA (sgRNA) target sequences of *EcIAG* were designed by the online tool CRISPRdirect (40). The sgRNA was synthesized according to the protocol reported from our laboratory (27) using the Thermo Scientific TranscriptAid T7 High Yield Transcription Kit (Thermo Fisher Scientific, USA). After synthesis, the sgRNA was purified by phenol-chloroform extraction. The concentration and quality were assessed by Nanodrop 2000 spectrophotometer (Thermo Fisher Scientific, Waltham, MA, USA) and 1% agarose gel electrophoresis, respectively.

### 2.6 Microinjection of *E. carinicauda* embryos

The embryos at the zygote stage or at the first cleavage were microinjected according to previous reported technique (29). Two sgRNAs and Cas9 protein (GenScript, Nanjing, China) were incubated at room temperature for 10 min to form Cas9 ribonucleoproteins (RNPs). Then, the RNPs were injected into the zygotes or the embryos at the first cleavage. The concentration of sgRNA and Cas9 protein were both 1000 ng/μl. Each fertilized egg was injected with approximately 0.5 nl RNPs complex. The microinjection was performed using standardized Femtotip II sterile microcapillaries (Eppendorf, Germany), FemtoJet 4i-Injector microinjector (Eppendorf, Germany) and stereomicroscope MMO4 micromanipulator (Narishige, Japan). After injection, every 500 embryos were hatched in a large petri dish with filtering seawater on a shaker at 100 rpm at room temperature. The filtering seawater was changed three times a day. When the fertilized embryos hatched, the mysis larvae of *E. carinicauda* were fed with anemia larvae. When the mysis larvae developed into juvenile prawns, they were fed with clams.

### 2.7 Evaluation on the gene editing efficiency

Five days after microinjection, a total of 344 embryos after blastocyst stage were randomly selected for gene editing efficiency evaluation, of which 229 embryos were microinjected at the zygote stage and 115 were microinjected at the first cleavage. Using the micro extraction method, we acquired the genomic DNA. Briefly, each embryo was cleaved at 95 °C for 10 minutes using the MightyPrep reagent for DNA (Takara, Kyoto, Japan). Subsequently, the lysate was centrifuged at 12000rpm for 10 minutes and the supernatant was absorbed as a template. Then, the target sequence (295bp) flanking the sgRNA target site was amplified by PCR using the specific primers (**Table 1**) with Golden polymerase (Tiangen, Beijing). Fragment amplified from wild type (WT) embryos was used as control. The PCR program was set as follows: 94 °C for 5 min; 35 cycles of 98 °C for 30 s, 57°C for 30 s, and 72 °C for 30 s; 72 °C for 10 min. The PCR products were first detected by 1% agarose gel electrophoresis, and then sent to Sangon Biotech Co., Ltd. (Shanghai, China) for Sanger sequencing. Editing efficiency was evaluated by sequence alignment and chromatograms detecting using MEGA version 7.0 and Chromas software (https://technelysium.com.au/wp/chromas/).

### 2.8 Detection on CRISPR/Cas9 generated mutants of *EcIAG*

When the juvenile developed to adult, one leg of the adult was cut for gene editing detection. The PCR amplification, agarose gel electrophoresis, sequencing, sequence alignment and sequencing chromatogram detecting were all performed as described above. Then, the PCR products of mutants were cloned into pMD-19T vector (TaKaRa, Kyoto, Japan) and transformed into the Tans5α chemically competent cell (TransGen Biotech, Beijing, China). The positive clones were sequenced and performed sequence alignment to determine the specific type of mutation. The sex phenotype of the mutant was detected manually under the Nikon 80i microscope according to whether they have petasma between the fifth pair of walking legs. The genetic sex of the mutant was identified by a DNA marker for genetic sex identification of *E. carinicauda*. Briefly, the DNA marker amplification primers (EcSDF/EcSDR) were used to amplify the DNA of the mutant by PCR using the Golden polymerase (Tiangen, Beijing). The PCR program was set as follows: 94 °C for 5 min; 35 cycles of 94 °C for 30 s, 60°C for 30 s, and 72 °C for 30 s; 72 °C for 10 min. Subsequently, the PCR product was sent to Sangon Biotech Co., Ltd. (Shanghai, China) for sanger sequencing. The chromatograms of SNPs were detected using Chromas software (https://technelysium.com.au/wp/chromas/). Then, the evaluation of mutants was conducted by comparing phenotypic sex and genetic sex. The DNA markers and primers used for genetic sex determination are derived from the patent “A DNA marker for Genetic sex determination of *E. carinicauda* and its application” with the patent application number 202310392890.4.

## 3 Results

### 3.1 Identification and characterization of *EcIAG* in *E. carinicauda*

**Figure 1:**
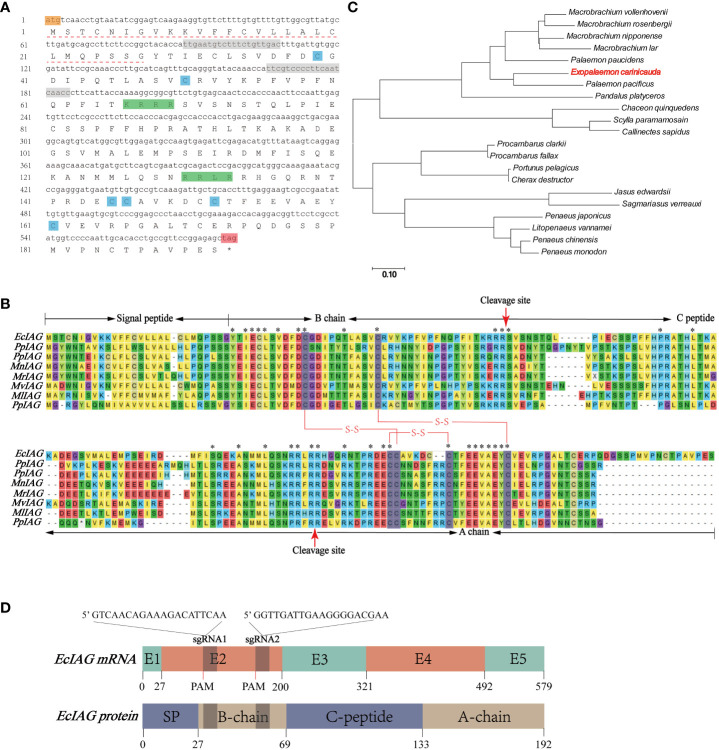
Sequences and structure information of *EcIAG* and sgRNA. **(A)** Nucleotide and deduced amino acid sequences of *EcIAG*. Nucleotide and deduced amino acid sequences of *EcIAG* were shown in lowercase and uppercase, respectively. The start codon ATG was marked with orange and the stop codon TAG was marked with red and indicated with “*”. The Signal peptide was marked with a dashed line. The conserved cysteine residues were marked with blue shading. The cleavage sites were marked with green shading. The two sgRNAs targeted site were marked with gray shading. **(B)** Multiple sequence alignment of *EcIAG* and IAG proteins from other crustaceans. The signal peptide, B chain, C peptide and A chain were marked. Six conserved cysteine residues were marked with gray shading, and three putative disulfide bridges were shown by red lines. The cleavage sites were marked with red arrows. Asterisks indicated fully conserved residues. The IAG sequences from *E*. *carinicauda* (*EcIAG* in this paper)*, Palaemon pacificus* (*PpIAG*, BAJ84109.1), *Palaemon pauciden* (*PpIAG*, BAJ84108.1), *Macrobrachium rosenbergii* (*MrIAG*, ACJ38227.1), *Macrobrachium nipponense* (*MnIAG*, AGB56976.1), *Macrobrachium vollenhovenii* (*MvIAG*, AHZ34725.1), *Macrobrachium lar* (*MlIAG*, BAJ78349.1), *Pandalus platyceros* (*PpIAG*, ASM94212.1) were used for alignment. **(C)** Phylogenic relationships of IAG homologs in decapod crustaceans. The phylogenic tree was constructed via MEGA7.0 using neighbor-joining (NJ) approach with 1,000 bootstrap replicates. The IAG sequences used in this study were from *Macrobrachium vollenhovenii* (AHZ34725.1), *Macrobrachium rosenbergii* (ACJ38227.1), *Macrobrachium nipponense* (AHA33389.1), *Macrobrachium lar* (BAJ78349.1), *Palaemon paucidens* (BAJ84108.1), *Palaemon pacificus* (BAJ84109.1), *Pandalus platyceros* (ASM94212.1), *Chaceon quinquedens* (ASA45642.1), *Scylla paramamosain* (AIF30295.1), *Callinectes sapidus* (AEI72263.1), *Procambarus clarkia* (ALX72789.1), *Procambarus fallax* (ASM94213.1), *Portunus pelagicus* (ADK46885.1), *Cherax destructor* (ACD91988.1), *Jasus edwardsii* (AIM55892.1), *Sagmariasus verreauxi* (AHY99679.1), *Penaeus japonicus* (BAK20460.1), *Litopenaeus vannamei* (AIR09497.1), *Penaeus chinensis* (AFU60546.1), *Penaeus monodon* (ADA67878.1) and *E*. *carinicauda* (EcIAG in this paper). **(D)**The putative linear structure of *EcIAG* mRNA and secondary structure of EcIAG protein. Length of exon are indicated with number. The mRNA and amino acid sequences were one-to-one correspondences and were marked with number of nucleic acids and amino acids. Two sgRNAs sequences were shown with base. The protospacer adjacent motif sites were marked with PAM.

By screening the genomic and transcriptomic data, a gene annotated as IAG (named *EcIAG*) was identified in *E. carinicauda*. The ORF of *EcIAG* is 579 bp and encodes 192 amino acids (NCBI accession: OR663996). The predicted molecular weight of EcIAG protein is 21.5 kDa, and the PI value is 7.43. The linear structure of EcIAG protein exhibits a similar structure to IAGs from other species, including a signal peptide at the N-terminal, followed by B chain, C peptide and A chain. Moreover, the amino acid sequence contains six conserved cysteines and two cleavage sites, KRRR and RRLR (**Figures 1A, B**). The two and four cysteines in B and A chains, respectively, was predicted to form two interchain and one intrachain disulfide bonds, which are important for the tertiary structure of mature peptide (**Figure 1B**). The EcIAG protein had the highest similarity with its homologue from *Palaemon pacificus* (64.41%), followed by *Palaemon paucidens* (57.23%), *M. rosenbergii* (50.29%), *Macrobrachium nipponense* (49.71%), *Macrobrachium vollenhovenii* (48.28%), *Macrobrachium lar* (45.86%) and *Pandalus platyceros* (40.54%) (**Table 2**). The sequences with high amino acid sequence similarity to *EcIAG* were all derived from *Palaemonidae*, except for *Pandalus platyceros.* IAGs from decapod species have highly conserved characteristics, including B chain, A chain, six conserved cysteine residues and two cleavage sites (**Figure 1B**). Phylogenetic tree analysis indicated that EcIAG was clustered with *Palaemonidae* homologs and separated from other IAG members of decapod crustaceans (**Figure 1C**), consistent with the sequence alignment results.

**Table 2:** The information of IAG protein used in multiple sequence alignment.

Species	GenBank No.	Description	Similarity
*Palaemon pacificus*	BAJ84109.1	insulin-like androgenic gland factor	64.41%
*Palaemon paucidens*	BAJ84108.1	insulin-like androgenic gland factor	57.23%
*Macrobrachium rosenbergii*	ACJ38227.1	insulin-like androgenic gland specific factor	50.29%
*Macrobrachium nipponense*	AGB56976.1	insulin-like androgenic gland factor	49.71%
*Macrobrachium vollenhovenii*	AHZ34725.1	insulin-like androgenic gland hormone	48.28%
*Macrobrachium lar*	BAJ78349.1	insulin-like androgenic gland factor	45.86%
*Pandalus platyceros*	ASM94212.1	insulin-like androgenic gland hormone	40.54%

### 3.2 Location of sgRNA

Mapping the ORF sequence to the genomic sequence indicated that *EcIAG* contained five exons and four introns (**Figure 1D**). Both sgRNA fragments target the second exon of *EcIAG*, which encodes part of signal peptide and the B chain (**Figure 1D**). The simultaneous operation of two sgRNA fragments would lead to a large deletion of the nucleotide sequence, which contains two conserved cysteine residues that form two interstrand disulfide bonds of the mature IAG peptide (**Figure 1A**).

### 3.3 The mutation rate and type of the CRISPR/Cas9 system

**Figure 2:**
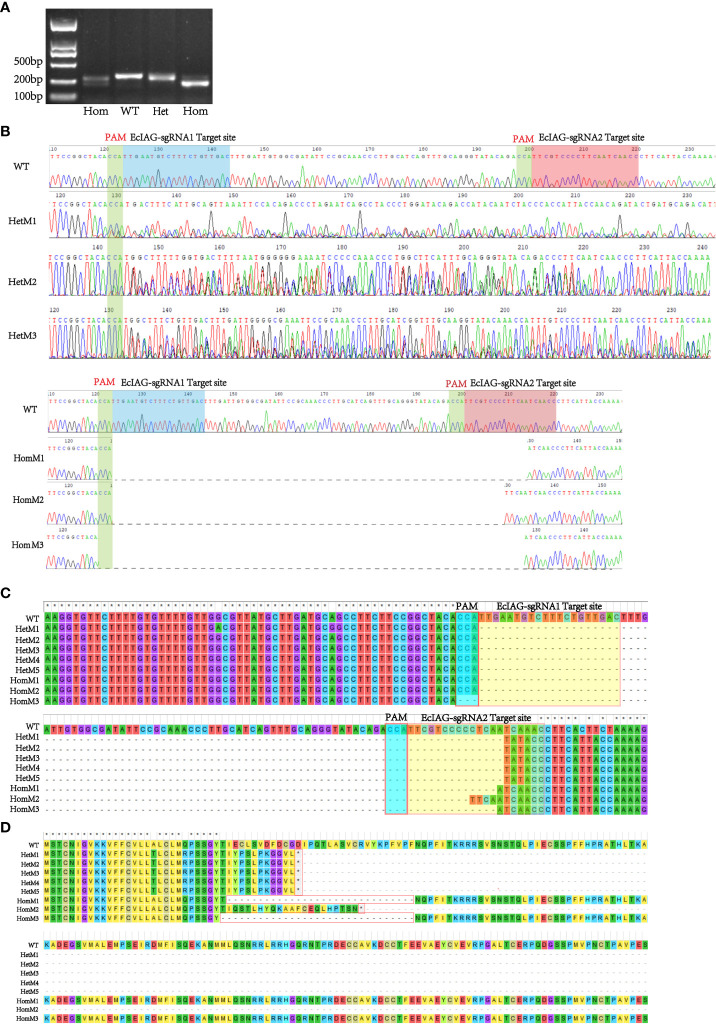
Detection of the *EcIAG* mutations of adult prawns. **(A)** Agarose electrophoresis analysis of wild-type (WT) and mutant (HetM and HomM) PCR products. The electrophoresis bands of HomM were shorter compared with WT and HetM. **(B)** The representative sequencing chromatograms of heterozygous and homozygous mutants. The top sequence represents the wild-type (WT) sequences. The protospacer adjacent motif (PAM) sites were indicated by green shading and the two sgRNAs targeted site were indicated by blue and red shading. **(C)** A large deletion occurred in the nucleotide sequence between the two sgRNA target sites. **(D)** Changes of nucleotide led the deletion of amino acid residues (HomM1 and HomM3) and the premature termination of translation (HetM1-5 and HomM2). The heterozygous mutant was indicated with HetM, the homozygous mutant was indicated with HomM.

The editing efficiency of Cas9 RNP was assessed in embryos after blastula stage. The sequencing data showed that the genome of *E. carinicauda* had been successfully edited in some embryos. The types of mutations could be divided into heterozygous mutation and homozygous mutation. The chromatogram of heterozygous mutant (HetM) showed multiple peaks at the protospacer adjacent motif (PAM) site, while the wild did not show (**Figure 2B**). However, in the homozygous mutant (HomM), there was no multiple peaks in the sequencing map, but the electrophoretic band and the sequence length were significantly shortened (**Figures 2A, B**). A total of 229 embryos injected at zygote stage were detected, of which 129 were amplified and sequenced successfully, and 29 were mutated, with an editing efficiency of 22.48%. Besides, a total of 115 embryos injected at the first cleavage, of which 86 were amplified and sequenced successfully, and 12 were mutated, with an editing efficiency of 13.95% (**Table 3**).

**Table 3:** Statistics on the editing efficiency of EcIAG mediated by CRISPR/Cas9 system.

Target gene	Injection		Editing efficiency		Hatching rate	Survival adult	Adult mutant	
EcIAG	Injected at zygote stage	Injected at the first cleavage	Injected at zygote stage	Injected at the first cleavage	(40/266) 15.04%	20	heterozygous mutant	homozygous mutant
	400	200	22.48%	13.95%			5	3

### 3.4 Phenotypic changes in *E. carinicauda* after gene editing

**Figure 3:**
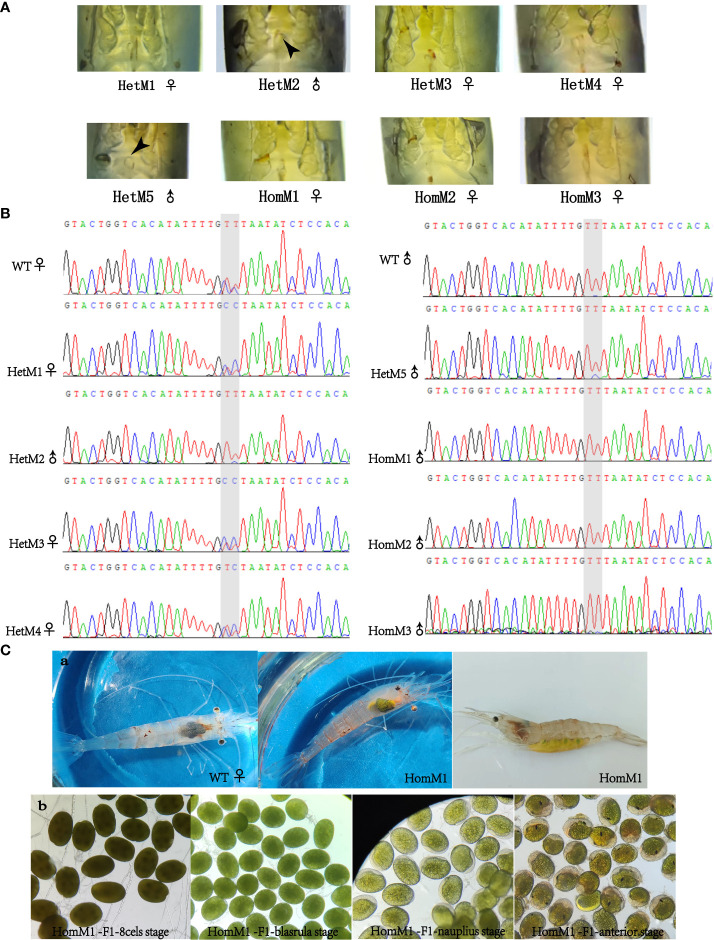
The phenotypic and genetic evaluation of *EcIAG* mutants acquired by CRISPR/Cas9 technology. **(A)** Phenotypic sex of mutants. The petasma of mutant with phenotypic male sex was marked with black arrow. **(B)** The sequencing chromatograms of sex DNA markers of mutants. For the two adjacent SNP markers marked with gray shading, male individuals were both T/T-T/T genotypes, and female individuals were both C/T-C/T genotypes. The males are indicated with “♂”, the females are indicated with “♀”. **(C)** The ovary development, oocytes production and embryonic development (8 cells stage, blastula stage, nauplius stage and anterior stage) of HomM1 mutant. The ovary was indicated with black arrow.

**Figure 4:**
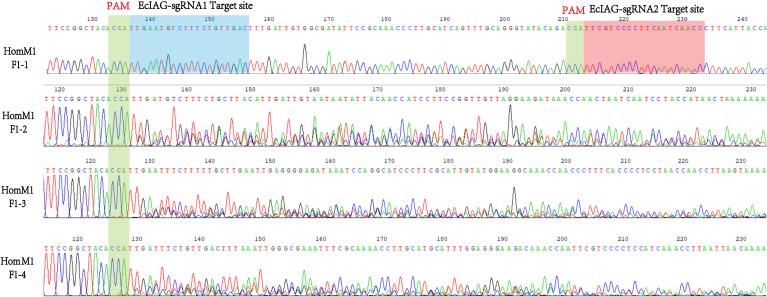
The representative sequencing chromatograms of HomM1 F1 generation. The top sequence represents the wild-type (WT) sequences. The protospacer adjacent motif (PAM) sites were indicated by green shading and the two sgRNAs targeted site were indicated by blue and red shading.

After microinjection, a total of 40 larvae hatched, and 20 prawns grew to adults. CRISPR/Cas9-mediated gene editing technology induced mutations in eight individuals, of which five were heterozygous mutants and three were homozygous mutants. The five heterozygous mutants occurred both deletion and insertion mutations, leading to premature termination of translation. The three homozygous mutants all had large deletions in nucleotide sequences, leading to large deletions in amino acid sequences and premature termination of translation (**Figures 2C, D**). In addition to assessing editing effects, we also checked the genetic and phenotypic and genetic sex of mutants (**Figures 3A, B**, **Table 3**). Among the five heterozygous mutants, the genetic sex of three was female and that of the other two was male, and the genetic sex was consistent with the sex phenotype (**Figures 3A, B**, **Table 4**). Three homozygous mutants with a genetic male sex lost their male characteristics and exhibited female characteristics (**Figures 3A–C**, **Table 4**). As development progresses, the ovary of HomM1 also developed normally. After crossing with a wild male, HomM1 could produces oocytes. And, the embryos developed normally and hatched (**Figure 3C**). Gene editing evaluation on the first filial generation (F1) showed that the *EcIAG* mutation could passed on to the offspring (**Figure 4**).These results indicated that sex reversal could achieve by knocking out *EcIAG*, and the mutation induced by CRISPR/Cas9 was hereditary.

**Table 4:** Phenotypic and genetic features of *E. carinicauda* mutants.

Mutants	Phenotype sex	Genetic sex
HetM1	Female	Female
HetM2	Male	Male
HetM3	Female	Female
HetM4	Female	Female
HetM5	Male	Male
HomM1	Female	Male
HomM2	Female	Male
HomM3	Female	Male

## 4 Discussions

In the present study, the male sexual differentiation gene *IAG* was obtained from *E. carinicauda*. Using CRISPR/Cas9-mediated gene editing technology, *EcIAG* was knocked out and homozygous mutants with biallelic mutations were acquired, achieving the sex reversal of *E. carinicauda* from males to neo-females. The study verified the role of *EcIAG* in the sexual differentiation of male *E. carinicauda* and acquired sex reversal in crustaceans for the first time using CRISPR/Cas9 system (11). The gene structure and function of IAGs are highly conserved in crustaceans (19, 29), so it might be possible to use CRISPR/Cas9-mediated gene editing technology to knock out IAG genes in other crustaceans and lead to sex reversal, which has importantly practical value in the monosex culture of crustaceans.

**Figure 5:**
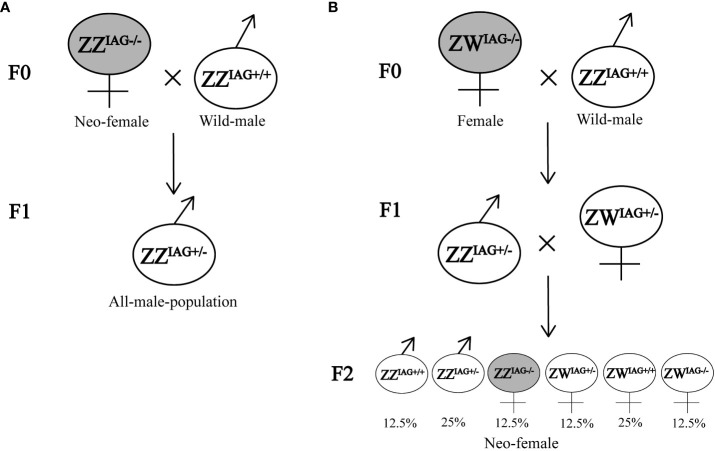
Potential crossing scheme of creating monosex population. **(A)** Neo-female with genotype ZZIAG-/- crossed with wild male. **(B)** Female with genotype ZWIAG-/- crossed with wild male and acquired neo-females in F2 generation. F0 represented the mutants and wild types. F1 represented the offspring of EcIAG knockout mutants crossed with wild types. F2 represented the offspring of F1 inbreeding.

Compared with existing methods of obtaining monosex populations, such as manual segregation, AG manipulation or silencing sexual differentiation related genes, CRISPR/Cas9-mediated gene editing technology has many advantages. It is efficient and labor-saving method, which does not need to consider the decision-making point of sexual differentiation or intervention time, and could obtain inheritable mutations (1, 20). In *M. rosenbergii*, although the fully functional sex reversal from males to females could be acquired by AG ablation or RNA interference, they were limited by the developmental stage of the prawn, and the mutation could not be inherited (7, 22). In our study, the mutation of *EcIAG* induced by gene editing could be passed on to offspring. We could expand the population of mutants by breeding, and create all-male or all-female populations by crossbreeding. For example, the genetic sex determination system of *E. carinicauda* was ZZ/ZW system, and the F1 generation would be all heterozygous male mutants (ZZ^IAG+/-^) (**Figure 5A**). This is the most efficient way to create a monosex male population without the need for constant mating. Moreover, if one homozygous female mutant (ZW^IAG-/-^) obtained through the gene editing system crosses with a wild male (ZZ ^IAG+/+^), we could acquire neo-females (ZZ^IAG-/-^) by free mating in F1 generation (**Figure 5B**).

Microinjection is the main workhorse to deliver CRISPR/Cas9 reagents for gene editing. The survival rate and gene editing efficiency of injected embryos and are two mainly limiting factors for the application of this technique in crustaceans (41). In previous studies, the efficiency of CRISPR/Cas9-mediated gene editing technology in crustaceans was generally around 10% and usually led to heterozygous mutants (29, 32, 42). In the present study, the gene editing efficiency in embryos of *E. carinicauda* reached 22.48% and the homozygous *EcIAG* mutants was acquired. The increasement of gene editing efficiency might be partially attributed to the injection of two sgRNA fragments, which has been reported to improve gene editing efficiency (26, 30). In addition, two sgRNAs could function not only in the zygotes but also in the embryo after its first cleavage. This may be related to the meroblastic cleavage of *E. carinicauda* zygotes in the early cleavage stage (43). Moreover, the using of Cas9 nuclease protein might also contribute to the high gene editing efficiency, since it is delivered in functional form, and it also has less toxicity compared with plasmid and mRNA (44, 45). And, the use of higher concentrations of sgRNA and Cas9 nuclease protein than those used in previous studies might also be the reason for the high efficiency of gene editing (28, 29, 46). However, although the editing efficiency has improved significantly, the survival rate of embryos is still low. We still need to optimize injection techniques to further improve the survival rate of embryos. With the development of technology, some alternative deliver approaches have been developed in other species, such as ReMOT Control, electroporation, nanoparticle delivery, etc (41, 47, 48). By using novel CRISPR/Cas9 reagents delivery methods, we might be able to create more efficient CRISPR/Cas9 gene editing system.

The in-depth study on the sexual differentiation of the IAG gene provides a basis for the use of CRISPR/Cas9 gene editing technology to achieve sex manipulation in crustaceans. In addition to *IAG*, several genes homologous to *Sxl*, *Tra-2*, *Dmrt* and *Dsx*, which are involved in the insect sexual differentiation cascade, have been identified from many crustaceans (49–53). These genes share high sequence similarity with their homologs in insects and their expression profiles have been characterized in crustaceans. However, few genes have been functionally studied as deeply as the *IAG* gene. Therefore, the detailed roles of these genes in sex determination and differentiation remain largely unknown in crustaceans. With the mature application of gene editing technology in crustaceans, the function of these genes will be illustrated and new targets for sex manipulation will also be developed.

## 5 Conclusion

In the present study, the function of *EcIAG* in male sexual differentiation was characterized and the sex reversal in *E. carinicauda* was obtained after knocking out *EcIAG* using the CRISPR/Cas9-mediated gene editing technology. Knockout of the target gene could be achieved by microinjection at both the zygote stage and the embryo at the first cleavage. Homozygous mutation of *EcIAG* in both chromosome sets directly led to the sex reversal of male individuals into neo-females. The present study provides not only a new method for sex manipulation and monosex population breeding, but also important experimental materials for studying the mechanism of sexual differentiation in crustaceans.

## Data availability statement

The datasets presented in this study can be found in online repositories. The names of the repository/repositories and accession number(s) can be found in the article/supplementary material.

## Ethics statement

Ethical approval was not required for the study involving animals in accordance with the local legislation and institutional requirements because this study used shrimp and experimental animals, which is not endangered invertebrates. In addition, there is no genetically modified organism used in the study. According to the national regulation (Fisheries Law of the People’s Republic of China), no permission is required to collect the animals and no formal ethics approval is required for this study.

## Author contributions

MM: Data curation, Formal Analysis, Validation, Visualization, Writing – original draft. SL: Conceptualization, Project administration, Supervision, Writing – review & editing. JY: Data curation, Funding acquisition, Investigation, Writing – review & editing. PL: Data curation, Investigation, Methodology, Writing – review & editing. XF: Investigation, Methodology, Writing – review & editing. CZ: Methodology, Writing – review & editing. XZ: Writing – review & editing. FL: Conceptualization, Funding acquisition, Supervision, Writing – review & editing.
